# Extensive White Matter Alterations and Its Correlations with Ataxia Severity in SCA 2 Patients

**DOI:** 10.1371/journal.pone.0135449

**Published:** 2015-08-11

**Authors:** Carlos R. Hernandez-Castillo, Victor Galvez, Roberto Mercadillo, Rosalinda Diaz, Aurelio Campos-Romo, Juan Fernandez-Ruiz

**Affiliations:** 1 Consejo Nacional de Ciencia y Tecnología—Cátedras—Instituto de Neuroetologia, Universidad Veracruzana, Mexico DF, Mexico; 2 Instituto de Neuroetologia, Universidad Veracruzana, Xalapa, Mexico; 3 Cátedras Consejo Nacional de Ciencia y Tecnología, Área de Neurociencias, Departamento de Biología de la Reproducción, Universidad Autónoma Metropolitana, Mexico DF, Mexico; 4 Laboratorio de Neuropsicología, Departamento de Fisiología, Facultad de Medicina, Universidad Nacional Autónoma de México, Mexico DF, Mexico; 5 Unidad Periferica de Neurociencias, Facultad de Medicina Universidad Nacional Autonoma de Mexico/Instituto Nacional de Neurología y Neurocirugía, Mexico DF, Mexico; Fraunhofer Institute for Cell Therapy and Immunology, GERMANY

## Abstract

**Background:**

Previous studies of SCA2 have revealed significant degeneration of white matter tracts in cerebellar and cerebral regions. The motor deficit in these patients may be attributable to the degradation of projection fibers associated with the underlying neurodegenerative process. However, this relationship remains unclear. Statistical analysis of diffusion tensor imaging enables an unbiased whole-brain quantitative comparison of the diffusion proprieties of white matter tracts in vivo.

**Methods:**

Fourteen genetically confirmed SCA2 patients and aged-matched healthy controls participated in the study. Tract-based spatial statistics were performed to analyze structural white matter damage using two different measurements: fractional anisotropy (FA) and mean diffusivity (MD). Significant diffusion differences were correlated with the patient's ataxia impairment.

**Results:**

Our analysis revealed decreased FA mainly in the inferior/middle/superior cerebellar peduncles, the bilateral posterior limb of the internal capsule and the bilateral superior corona radiata. Increases in MD were found mainly in cerebellar white matter, medial lemniscus, and middle cerebellar peduncle, among other regions. Clinical impairment measured with the SARA score correlated with FA in superior parietal white matter and bilateral anterior corona radiata. Correlations with MD were found in cerebellar white matter and the middle cerebellar peduncle.

**Conclusion:**

Our findings show significant correlations between diffusion measurements in key areas affected in SCA2 and measures of motor impairment, suggesting a disruption of information flow between motor and sensory-integration areas. These findings result in a more comprehensive view of the clinical impact of the white matter degeneration in SCA2.

## Introduction

Spinocerebellar ataxias (SCAs) are a group of clinically and genetically heterogeneous autosomal dominant neurodegenerative diseases characterized by a range of neurological symptoms, including loss of balance and motor coordination. The primary cause of SCAs is by the progressive dysfunction of the cerebellum and of its afferent and efferent connections [1]. Spinocerebellar Ataxia Type 2 (SCA2) is caused by an expanded CAG trinucleotide repeat in the gene ATXN2 encoding the protein ataxin-2 [2]. It is characterized by a progressive cerebellar syndrome including ataxic gait, cerebellar dysarthria, dysmetria, dysdiadochokinesia and other visuospatial impairments including saccadic and voluntary eye movements [3–5]. Several neuropathological studies have revealed a generalized reduction in brain volume, with significant atrophy of the cerebellum, brainstem, and frontal lobe, as well as changes to the midbrain substantia nigra and reduction of the cerebral and cerebellar white matter (WM)[6]. A number of studies using magnetic resonance imaging (MRI) to explore the SCA2 neurodegenerative process have confirmed olivopontocerebellar atrophy, and in some cases significant degeneration of the thalamus and cortical areas [7–9]. Furthermore, a recent longitudinal study has even suggested the use of MRI as a possible biomarker of the SCA2 degenerative process [10].

Advances in MRI, particularly diffusion tensor imaging (DTI), allow the acquisition of detailed structural images in a millimetric resolution, reflecting the tissue microstructure and integrity through the measurement of water diffusion properties [11]. DTI enables mapping white matter tract changes across the life span, as well as alterations in neurological disorders, becoming an important tool in the study of neurodegenerative diseases [12]. Mean diffusivity (MD) (also referred to as apparent diffusion coefficient, ADC) and fractional anisotropy (FA) have gained widespread acceptance as sensitive indicators to quantify microstructural damage of gray and white matter in neurodegenerative diseases including SCAs [13–16]. Although a number of studies have analyzed WM changes using these methods in SCA2 [17–19], only one study has found correlation between white matter integrity and clinical scores changes using a global analysis in SCA2. This earlier study acquired DTI images in 15 directions of 10 SCA2 patients using a 1.5T MRI scanner, and found correlations between the International Cooperative Ataxia Rating Scale (ICARS) scores and MD values in the left cerebellar hemisphere and in the left fornix [18].

To further understand the relationship between WM integrity and SCA2’s ataxia severity, we assessed 14 patients with SCA2 and age-matched controls using a voxel-wise whole-brain analysis of multi-subject diffusion tensor data named Tract-Based Spatial Statistics (TBSS) [20,21]. The resulting significant group differences in WM were correlated with the measures of ataxia impairment using the Scale for Assessing and Rating Ataxia (SARA). We found significant WM group differences across the brain, including WM alterations that showed significant correlations with the SARA score not previously reported.

## Materials and Methods

### Subjects

14 patients with a molecular diagnosis of SCA2 were invited to participate in this study (9 female, right handed, mean age ± SD, 37.3 ± 15.9 years, complete information in S1 Table).The SARA [21] was used as a semi-quantitative valuation of the movement impairment comprising eight items related to gait, stance, sitting, speech, finger-chase test, nose-finger test, fast alternating movements, and heel-shin test [22,23]. 14 controls (8 female, right handed, mean age 41.7 years) participated in the study. The control group denied any history of neurological or psychiatric disorders. All procedures were in accordance with the ethical standards of the responsible committee on human experimentation (institutional and national),with the Helsinki Declaration of 1975, and the applicable revisions at the time of the investigation. Therefore, the committees on human experimentation of the Universidad Nacional Autónoma de Mexico specifically approved this study. All participants gave their written informed consent before entering the study.

### Image Acquisition

Images were acquired using a 3 Tesla Philips Achieva MRI scanner (Philips Medical Systems, Eindhoven, The Netherlands). The study included the acquisition of a high resolution T1 3D volume and diffusion tensor imaging (DTI). The T1 3D acquisition consisted of a T1 Fast Field-Echo sequence, with TR/TE = 8/3.7 ms, FOV 256x256 mm^2^ and an acquisition and reconstruction matrix of 256x256, resulting in an isometric resolution of 1x1x1 mm3. The DTI sequences consisted of Single Shot Echo Planar Imaging sequences, acquiring 33 volumes of 70 axial slices (2 mm slice thickness and no separation), one for each of the 32 independent directions of diffusion with b = 800 s/mm^2^ and one corresponding to b = 0 s/mm^2^, TR/TE = 8467/60 ms, FOV 256x256 mm^2^ and an acquisition and reconstruction matrix of 128x128, resulting in an isometric resolution of 2x2x2 mm^3^.

### Voxel-Based Morphometry Analysis

For reference of gray matter atrophy, voxel-based morphometry (VBM) analysis [24] was performed using FSL (FMRIB, Oxford University, Oxford, UK) [25]. The VBM analysis closely followed that previously reported [26] and included seven steps: reorientation according to the antero-posterior commissure line, template creation to improve brains segmentation, normalization, segmentation in 3 classes of tissue (GM, WM and CSF), modulation, smoothing with a 2 mm full width half-maximum Gaussian kernel, and voxelwise statistical analysis [27].

### Diffusion Tensor Analysis

The DTI images were processed using FSL's Diffusion Toolbox [25]. Eddy current effects were corrected and the diffusion tensor model was adjusted to generate the fractional anisotropy maps for each participant. The statistical analysis was done in a voxel-wise manner using the TBSS methodology [20]. TBSS was done using the following steps: identification of a common registration target and alignment of all participants FA images to this target, creation of a mean FA map using the mean of all aligned FA images and of a thresholded skeletonized mean FA image, and projection of each participants FA image onto the skeleton and voxel-wise statistical analysis across subject on the skeleton-space FA data. Using the same nonlinear registration, skeleton and skeleton projection vectors derived from the FA analysis, MD data were projected onto the skeleton before voxel-wise statistical analysis across subjects [21]. A two-sample t-test between SCA2 and control group was performed for FA and MD independently using FSL's randomise [28]. Age was included as covariates of no interest. Correction for multiple comparisons was assessed using randomized permutation methods [28,29]. Only those voxels surviving this correction at a p value < 0.05 were considered as showing a significant group difference. The final parametric maps were parcellated, binarized, and labeled using the white matter atlas made at Johns Hopkins University [30] and the automated anatomical labeling atlas [31], and used as masks for further analysis. The standardized and skeletonized FA and MD images were then loaded in MATLAB R2014a (The Mathworks, Inc., Natick,MA) and using the parcellation masks, the individual FA and MD values were extracted. For each white matter region the mean FA and MD value of non-zero voxels were calculated. Pearson's correlation between SARA score and diffusion measurements were calculated and significant correlations were set at the p value < 0.05 after correcting for multiple comparisons using the false discovery rate method [32].

## Results and Discussion

VBM analysis showed a high degree of gray matter atrophy in patients with SCA2 compared with healthy controls (S1 Fig). Reductions in gray matter were found in the cerebellum, vermis, pons, and insular, frontal, parietal and temporal cortices.

**Fig 1:**
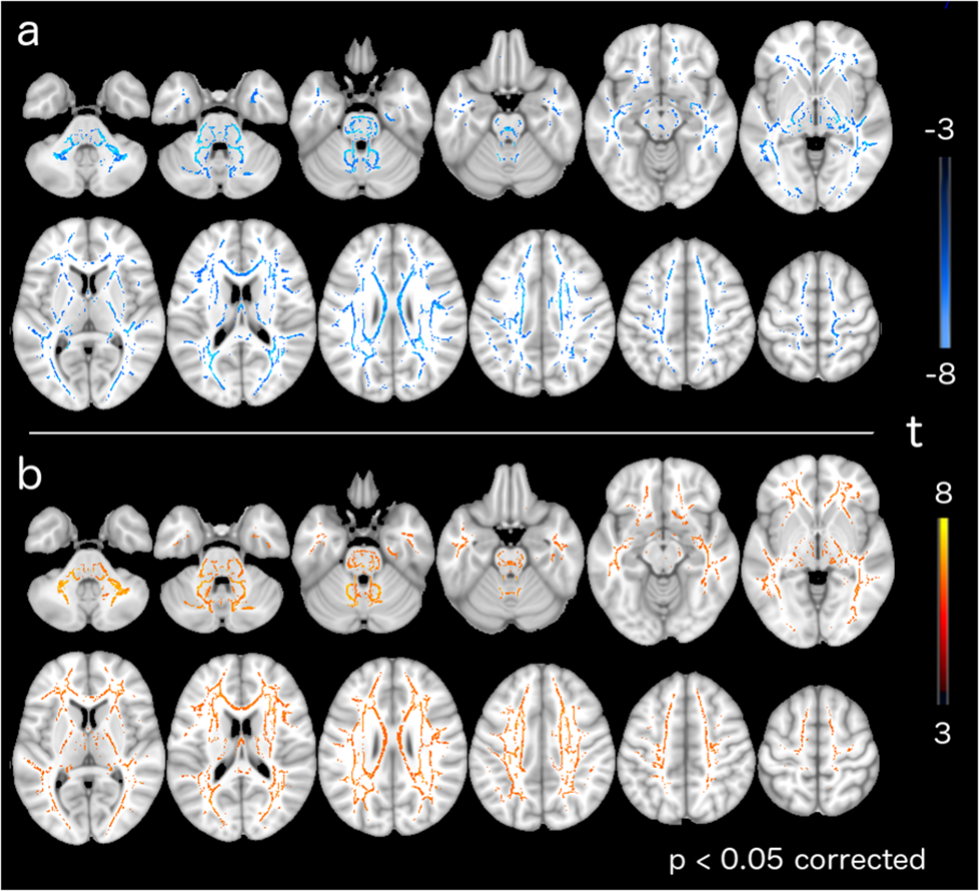
TBSS significant differences in diffusion measurements between SCA2 and healthy controls. a) Fractional anisotropy. b)Mean diffusivity. Warm and cold colors indicate an increase and decrease of these measures in the patients with SCA2, respectively.

TBSS group comparison revealed significant FA decreases in patients with SCA2 (Fig 1A) in white matter tracts including the inferior/middle/superior cerebellar peduncles, the bilateral posterior limb of the internal capsule, the bilateral superior corona radiata, the right posterior thalamic radiation and the medial lemniscus (for a complete list see S2 Table).

TBSS group comparison revealed significant MD increases in patients with SCA2 (Fig 1B) in cerebellar WM, including the medial lemniscus, the middle cerebellar peduncle, the bilateral anterior corona radiata, the posterior limb of internal capsule, the pontine crossing tract and the right corticospinal tract (for a complete list see S3 Table).

**Fig 2:**
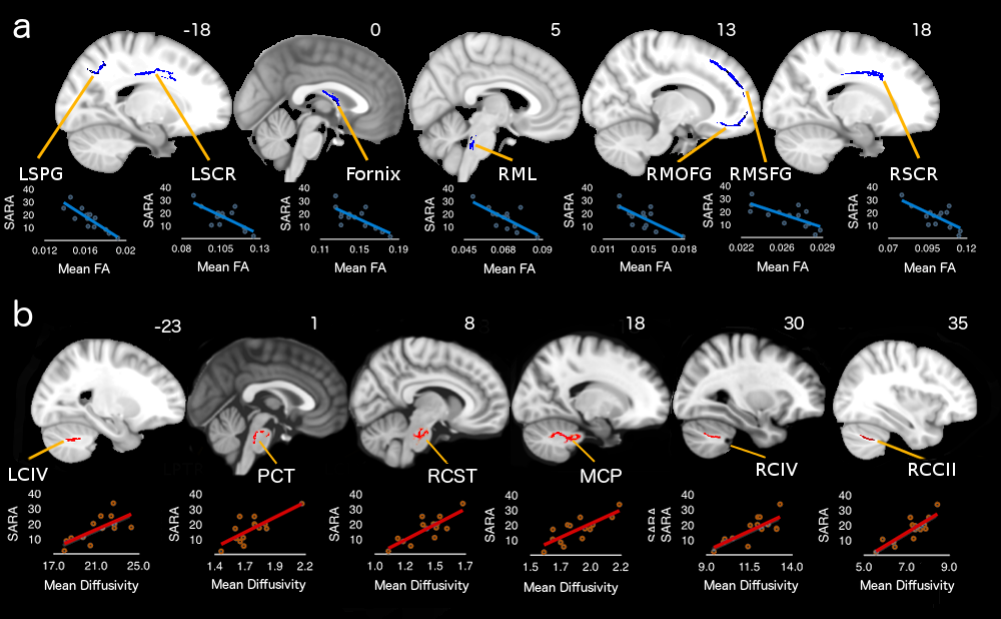
Significant correlations between abnormal diffusivity measurements and SARA scores. a) Fractional anisotropy. b) Mean diffusivity. For r and p values see Table 1. LSPG = left superior parietal gyrus; LSCR = left superior corona radiata; RML = right medial lemniscus; RMOFG = right medial orbitofrontal gyrus; RMSFG = right medial superior frontal gyrus; RSCR = right superior corona radiata; LCIV = left cerebellum lobule IV; PCT = Pontine crossing tract; RCST = right corticospinal tract; MCP = middle cerebellar peduncle; RCIV = right cerebellum lobule IV; RCCII = right cerebellum crus II.

SARA scores correlated with diffusion measurements in several significant abnormal WM tracts. Specifically, we found SARA correlations with FA in the parietal superior WM, the bilateral anterior corona radiata, fornix and the medial frontal gyrus WM (Fig 2A). Correlations with MD were found in cerebellar WM including bilateral lobule IV and crus I, the middle cerebellar peduncle, the pontine crossing tract and the right corticospinal tract (Fig 2B). Pearson's r and p values are detailed in Table 1 and Table 2.

**Table 1:** Regions showing correlation between fractional anisotropy and SARA score.

Anatomical region	r	p
Left Superior Parietal Gyrus	-0.85	0.0000
Left Superior Corona Radiata	-0.73	0.0027
Fornix	-0.73	0.0028
Right Medial Lemniscus	-0.76	0.0029
Right Medial Orbital Frontal Gyrus	-0.72	0.0031
Right Superior Corona Radiata	-0.72	0.0035
Right Medial Superior Frontal Gyrus	-0.71	0.0044

**Table 2:** Regions showing correlation between mean diffusivity and SARA score.

Anatomical region	r	p
Right Cerebellum Crus I	0.84	0.0001
Middle Cerebellar Peduncle	0.80	0.0004
Right Corticospinal Tract	0.78	0.0008
Right Cerebellum Lobule IV	0.76	0.0013
Pontine Crossing Tract	0.76	0.0014
Left Cerebellum Lobule IV	0.70	0.0046

## Discussion

Here we explored the relationship between WM areas found to be altered in SCA2 compared to controls, and SARA scores in a group of patients with SCA2. Our results showed significant deterioration in FA and MD measures not previously reported, including superior parietal WM and bilateral anterior corona radiata, as well as, cerebellar WM and middle cerebellar peduncle that correlated with the SARA clinical scores, respectively.

Our results also corroborated previous findings showing significant degeneration in the cerebellar peduncles, cerebellar WM, corona radiata and longitudinal fasciculus [17,18]. The expanded results obtained in the current study are probably due to the use of a higher magnetic field, larger number of directions, a different Ataxia rating scale, as well as a larger number of patients with SCA2.

As expected, TBSS analysis of FA and MD maps yielded only partially overlapping results. Several studies have shown that FA and MD are not equivalent measurements [33,34], while other studies including SCA1, SCA2 and Friedreich's ataxia participants have reported differences in diffusion metrics, including FA and MD, as well as, axial diffusivity(AD) and radial diffusivity(RD) [14,17,18]. In this work we focus on the analyses of FA and MD since AD and RD are subcomponents of MD, and to date, there is no consensus on if AD and RD are more useful or accurate in characterizing diffusion properties. Furthermore, several studies have suggested that MD is more sensitive and useful in the study of neurodegeneration than FA [12,35]. This debate, however, is beyond the scope of our current study. Therefore here we will focus on the discussion of the possible effects of the abnormalities found with both MD and FA.

### MD Correlations with SARA Score

The most significant correlation between SARA and MD was found in the WM of the cerebellar Crus I, follow by the middle cerebellar peduncle and the corticospinal tract. This result was expected based on the distribution of the neuropathological changes in SCA2 [5]. It is well known that lesions in these regions produce motor incoordination and loss of movement dexterity [36]. These regions were also reported as degenerated in previous studies [7,18]. However, in previous reports no significant correlations were found between the degree of the degeneration and SARA scores.

### FA Correlations with SARA Score

The most significant correlation between FA and SARA score was found in superior parietal WM. This region also showed gray matter atrophy (S1 Fig), so, the FA decreases were not unexpected. Sensory input and motor output signals are integrated in the superior parietal lobe to provide an internal estimate of the state of the body and the world [37], explaining why lesions in this region lead to both sensory and motor deficits [38]. FA in the bilateral superior corona radiata also correlated with SARA scores. The corona radiata is a group of fibers passing through the internal capsule projecting to the entire cerebral cortex. Corona radiata infarcts have been associated with ataxic-hemiparesis, which can also be found with other lesions of the corticopontine pathways [39,40]. Motor deficits have also been associated with the cortico-spinal tract after infarcts of the corona radiata that result in motor deficits related to the lesion severity [41]. In the same way, FA values in the medial lemniscus showed a correlation with SARA. The medial lemniscus connects the brain stem and the thalamus carrying information about touch, vibration and proprioception [42]. Medial lemniscus lesions have also been found in Friedreich ataxia, contributing to the deficits presented by these patients [43]. Another region where FA correlated with SARA was the fornix. While its exact function in the physiology of the brain is still not entirely clear, it has been demonstrated that surgical transection can cause spatial and visuomotor deficits [44,45]. Frontal cortex FA values correlated with SARA scores in the WM of the medial part of the superior frontal gyrus and in the orbitofrontal gyrus. Modulation of the superior frontal gyrus has been related to sensorimotor processing [46], and has also been associated with cognitive deficits in SCA6 [47]. The orbitofrontal gyrus shares extensive connections with other association cortices, including extensive local projections to and from other prefrontal regions, as well as with motor, limbic, and sensory cortices [46]. Its projections to motor areas are densely interconnected with other prefrontal cortical regions, reflecting integration for executive motor control [48]. The failure in the communication between frontal cortices and motor and sensory integration areas may impact the motor performance in this group of patients, as suggested by the relationship between ataxia deficits and the functional connectivity disruptions within the cerebellum and between the cerebellum and motor/parietal/frontal cortices in SCA2 [9] and in SCA7 [49,50].

## Conclusion

In conclusion, our study indicates that specific WM degenerative changes in SCA2 correlate with the severity of the ataxia. The degenerated tracts, where the diffusivity proprieties correlate with SARA scores, suggest a disruption of information flow between motor and sensory-integration areas. These findings contribute to a better understanding of the neural basis of the symptomatology presented by patients with SCA2.

## Supporting Information

S1 Fig Brain regions showing significant gray matter atrophy in SCA2 group. VBM differences between SCA2 and healthy controls. Warm colors indicate the degree of volume difference.
(PDF)

S1 Table Demographic information. (PDF)

S2 Table White matter regions showing significant FA decreases in SCA2. (PDF)

S3 Table White matter regions showing significant MD increases in SCA2. (PDF)
