# Supplementary figures and images for: Extensive White Matter Alterations and Its Correlations with Ataxia Severity in SCA 2 Patients
Source: PLoS One. 2015 Aug 11;10(8):e0135449. doi: 10.1371/journal.pone.0135449 (PMC4532454; doi:10.1371/journal.pone.0135449)

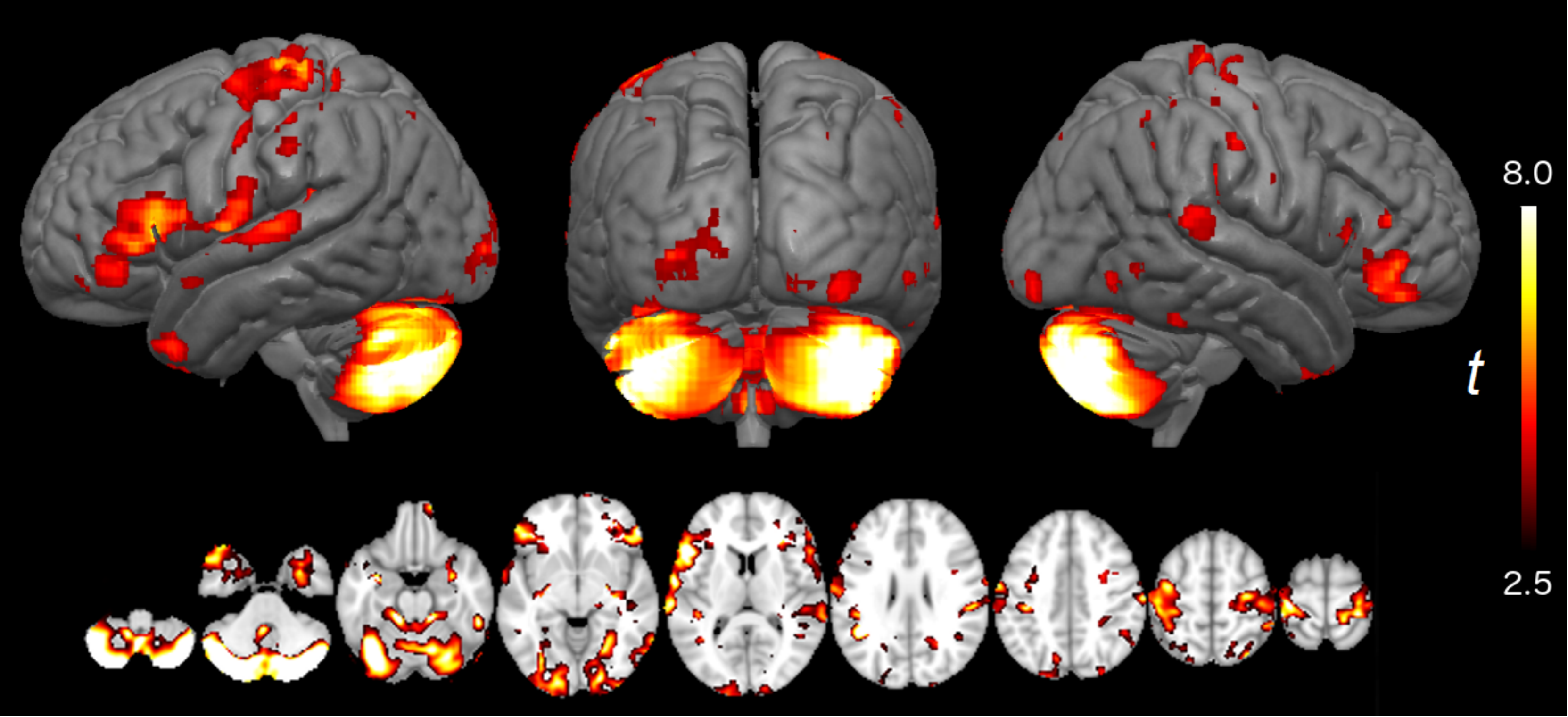

Supplement: S1 Fig — VBM differences between SCA2 and healthy controls. Warm colors indicate the degree of volume difference. (PDF) [file pone.0135449.s001.pdf]
