# Supplementary material for: Extensive White Matter Alterations and Its Correlations with Ataxia Severity in SCA 2 Patients
Source: PLoS One. 2015 Aug 11;10(8):e0135449. doi: 10.1371/journal.pone.0135449 (PMC4532454; doi:10.1371/journal.pone.0135449)
Supplement: S1 Table — (PDF) [file pone.0135449.s002.pdf]

Table S1. Demographic Information

ID	Age	Age at onset	CAG	SARA
P01	58	14	37	17.5
P02	31	25	42	17
P03	29	25	42	20
P04	20	14	45	10.5
P05	65	45	40	25
P06	60	42	39	20
P07	35	30	45	8.5
P08	29	25	51	27
P09	20	15	52	19.5
P10	46	36	42	17
P11	43	28	46	25
P12	19	19	42	5.5
P13	48	46	38	11
P14	41	18	48	33.5
