# Supplementary material for: Extensive White Matter Alterations and Its Correlations with Ataxia Severity in SCA 2 Patients
Source: PLoS One. 2015 Aug 11;10(8):e0135449. doi: 10.1371/journal.pone.0135449 (PMC4532454; doi:10.1371/journal.pone.0135449)
Supplement: S2 Table — (PDF) [file pone.0135449.s003.pdf]

Supplementary Table S2. White matter regions showing significant FA decreases in SCA2.

**Anatomical Region**	**X**	**Y**	**Z**	**t**
Middle Cerebellar Peduncle	2	-16	-26	-16.1
Right Inferior Cerebellar Peduncle	11	-44	-34	-11.5
Right Posterior Limb of Internal Capsule	18	-18	-3	-8.3
Right Superior Corona Radiata	31	-8	23	-8.3
Right Posterior Thalamic Radiation	29	-68	14	-8.1
Left Temporal Lobe	-45	-42	14	-7.9
Left Posterior Limb of Internal Capsule	-21	-20	-3	-7.8
Right Posterior Cerebellum Tonsil	32	-60	-37	-7.1
Right Temporal lobe	45	-40	-2	-6.9
Right Medial Lemniscus	5	-35	-31	-6.2
Right Retrolenticular part of Internal Capsule	36	-33	6	-5.9
Left Superior Longitudinal Fasciculus	-39	-23	30	-5.9
Right Medial Frontal Gyrus	15	27	44	-5.7
Right Superior Corona Radiata	18	-9	38	-5.7
Left Frontal Lobe	-30	35	3	-5.2
Left Superior Cerebellar Peduncle	-9	-52	-31	-5.2
Left Medial Frontal Gyrus	-14	21	46	-4.9
Right Superior Longitudinal Fasciculus	43	-13	29	-4.9
Left Superior Corona Radiata	-18	10	33	-4.8
Fornix	0	-4	14	-4.5
Left Superior Parietal Lobe	-17	-60	43	-4.4
Right Medial Frontal Gyrus	13	54	-11	-4.1

Coordinates in MNI space in millimeters. Highlighted rows indicates white matter regions showing correlation between FA and SARA.
