# Supplementary material for: Extensive White Matter Alterations and Its Correlations with Ataxia Severity in SCA 2 Patients
Source: PLoS One. 2015 Aug 11;10(8):e0135449. doi: 10.1371/journal.pone.0135449 (PMC4532454; doi:10.1371/journal.pone.0135449)
Supplement: S3 Table — (PDF) [file pone.0135449.s004.pdf]

Supplementary Table S3. White matter regions showing significant MD increases in SCA2.

| **Anatomical region** | **X** | **Y** | **Z** | **t** |
|---|---|---|---|---|
| Left Anterior Cerebellum Dentate | -15 | -56 | -29 | 12.9 |
| Right Medial Lemniscus | 5 | -35 | -35 | 11.5 |
| Middle Cerebellar Peduncle | 23 | -47 | -38 | 8.6 |
| Right Superior Corona Radiata | 27 | 12 | 24 | 8.27 |
| Right Posterior Cerebellum Tonsil | 30 | -63 | -36 | 7.9 |
| Left Temporal Lobe | -45 | -10 | -18 | 7.16 |
| Right Posterior Limb of Internal Capsule | 22 | -18 | -3 | 6.8 |
| Pontine Crossing Tract | 0 | -32 | -35 | 6.8 |
| Middle Cerebellar Peduncle | -17 | -46 | -33 | 6.14 |
| Right Temporal Lobe | 41 | -13 | -15 | 6.05 |
| Splenium of Corpus Callosum | 16 | -38 | 10 | 6.03 |
| Right Corticospinal Tract | 9 | -20 | -26 | 5.9 |
| Right Anterior Corona Radiata | 17 | 37 | 1 | 5.84 |
| Left Middle Temporal Lobe | -49 | -41 | -5 | 5.65 |
| Pontine crossing Tract | 1 | -32 | -35 | 5.55 |
| Left Precentral Gyrus | -40 | -1 | 30 | 5.12 |
| Right Coritcospinal Tract | 9 | -24 | -29 | 5.12 |
| Genu of Corpus Callosum | -3 | 22 | -1 | 4.79 |
| Right Superior Longitudinal Fasciculus | 43 | -24 | 31 | 4.7 |
| Left Anterior Corona Radiata | -14 | 33 | -8 | 4.6 |
| Left Corticospinal Tract | -8 | -20 | -25 | 4.12 |

Coordinates in MNI space in millimeters. Highlighted rows indicates white matter regions showing correlation between MD and SARA.
